# HistoNav: AI-Based H&E Histopathology Predicts Disease-Specific Survival and Guides Adjuvant Chemotherapy Decisions in Stage II/IIIA Colorectal Cancer

**DOI:** 10.3390/cancers18172863

**Published:** 2026-09-04

**Authors:** Xianhong Xu, Susan Fotheringham, Surya Rajan, Jamil Aliyev, David J. Kerr

**Affiliations:** 1 Oxford Cancer Biomarkers, The Magdalen Centre, Oxford Science Park, Robert Robinson Avenue, Oxford OX4 4GA, UK; kevin.xu@oxfordbio.com (X.X.); susan.fotheringham@oxfordbio.com (S.F.);; 2 Medical School, University of Birmingham, Birmingham B15 2T, UK; 3 Radcliffe Department of Medicine, University of Oxford, Oxford OX3 9DS, UK

**Keywords:** colorectal cancer, digital pathology, deep learning, disease-specific survival, risk stratification, prognostic biomarker, vision transformer, graph neural network

## Abstract

**Background**: Stage II/IIIA colorectal cancer (CRC) patients have a relatively high 5-year overall survival rate after surgical resection alone, but more than 50% of patients receive adjuvant chemotherapy. This study investigates a technology that applies artificial intelligence (AI) to conventional histopathology images to identify patients at risk of recurrence. **Methods**: HistoNav, a novel AI deep-learning algorithm based on Vision Transformer, Graph Neural Networks and Convolutional Neural Networks was designed to analyse H&E-stained formalin-fixed paraffin-embedded (FFPE) samples (*n* = 2095) to stratify Stage II/IIIA CRC patients into low-, intermediate-, and high-risk groups. **Results**: Data analysis revealed a 5-year disease-specific survival (DSS) of 93.2%, 84.3%, and 69.3% for low-, intermediate-, and high-risk groups, respectively. The hazard ratio for the high versus low-risk group (HR = 4.611, 95% CI: 2.776–7.662; *p* < 0.000001) was statistically significant, demonstrating HistoNav’s potential to stratify patients based on recurrence risk. **Conclusions**: HistoNav can effectively identify CRC patients with good prognosis using the digital images of H&E-stained resection samples and will support clinical decisions around the use of adjuvant chemotherapy.

## Simple Summary

More than 50% of Stage II/IIIA colorectal cancer patients receive adjuvant chemotherapy after surgical resection, despite a relatively high 5-year overall survival rate with surgery alone, and current staging cannot reliably identify which patients are truly low risk. Accurate preoperative risk stratification is therefore important for guiding adjuvant treatment decisions. We aimed to assess whether an artificial intelligence model applied to routine haematoxylin and eosin (H&E) whole slide images could stratify disease-specific survival risk in Stage II/IIIA colorectal cancer without additional molecular testing. Using 2095 patients across training and independent external validation cohorts, we developed HistoNav, which classified patients into low-, intermediate-, and high-risk groups with 5-year disease-specific survival of 93%, 84%, and 69% respectively (hazard ratio 4.611, high versus low risk; *p* < 0.000001). The high negative predictive value (0.961) for low-risk classification was consistent across independent cohorts despite differing scanning and staining protocols. These findings suggest H&E-based deep learning could offer a low-cost approach to safely de-escalate adjuvant chemotherapy in appropriately selected patients.

## 1. Introduction

Colorectal cancer (CRC) is the third most common cancer and the second leading cause of cancer-related death worldwide. With 1.93 million new diagnoses in 2020 alone, accounting for 10% of all cancer diagnoses [1], the imperative for better patient risk stratification is clear. However, the efficacy of post-surgery treatments such as adjuvant chemotherapy for stage II and IIIA CRC patients is often uncertain due to the biological and clinical heterogeneity of early-stage CRC [2]. Stage II CRC patients, in particular, have a relatively high 5-year overall survival rate after surgical resection alone (approximately 80%), the remaining 20% comprises approximately 4% who would benefit from adjuvant chemotherapy and 16% who are unlikely to benefit [3]. Notably, some Stage IIIA patients have a more favourable prognosis than Stage IIB or IIC patients, reflecting heterogeneity within conventional staging categories [4]. There is therefore a clear clinical need for prognostic tools capable of risk-stratifying patients beyond conventional staging criteria. Such tools would enable more personalised treatment decisions, sparing low-risk patients from unnecessary chemotherapy toxicity while directing treatment to those most likely to benefit.

Current clinical risk assessment relies on clinicopathological features to estimate recurrence risk. These include TNM stage, resection margin and invasion status, lymph node yield, and MMR/MSI status. While these factors inform broad treatment decisions, none reliably identifies individual patients who will benefit from adjuvant chemotherapy. Several technologies have emerged to improve prognostic precision beyond conventional staging, including liquid biopsy, molecular profiling, and artificial intelligence (AI)-based digital pathology [5].

By analysing circulating tumour cells (CTCs) [6] and circulating tumour DNA (ctDNA) [7,8] in the bloodstream, these assays offer a minimally invasive means of monitoring tumour burden and detecting minimal residual disease (MRD). ctDNA assays, in particular, have gained attention for their sensitivity in detecting molecular alterations and recurrence risk, even in the absence of radiological evidence. Studies have demonstrated that the presence of ctDNA after surgery or treatment is associated with a significantly higher risk of relapse across various cancer types, including colorectal, breast, and lung cancers [9].

In parallel, histopathology image-based prognostic models have emerged as powerful tools for recurrence risk prediction. These methods [10,11,12] leverage digitised whole slide images (WSIs) of stained tissue samples—most commonly haematoxylin and eosin (H&E)—to extract morphological and spatial features that are often difficult for human observers to quantify. These features are considered to correspond to the molecular, cellular, and physical changes in the tumour microenvironment (TME) instigated by the tumour cells and suppressed by the immune cells, such as myeloid-derived suppressor cells (MDSCs), macrophages, and tumour-infiltrating lymphocytes (TILs) within the stroma [13,14]. Deep learning models, particularly convolutional neural networks (CNNs) and vision transformers, have demonstrated strong performance in identifying prognostically relevant patterns within the TME. More recently, large-scale self-supervised foundation models trained across diverse tissue types have further expanded the representational capacity available for downstream prognostic tasks in computational pathology [15]. These approaches offer a non-destructive, data-rich, and scalable solution for risk stratification, complementing traditional molecular assays such as genomic sequencing or liquid biopsies [16].

Building on these modalities, multimodal deep learning models [17] have recently been developed to integrate histological and genomic data. These models combine WSI-derived morphological features with molecular profiles—such as gene expression, somatic mutations, and DNA methylation—to capture a more comprehensive view of tumour biology. Transformer-based and graph neural network (GNN) architectures are often employed to align these heterogeneous data types and uncover patterns predictive of survival outcomes [18]. In CRC, such integrative approaches have demonstrated improved predictive performance over single-modality models, enabling more personalised and biologically grounded prognostic assessments [19].

Compared with genomic or liquid biopsy-based approaches, histopathology image-based prognostic models offer a more cost-effective and scalable solution, particularly in settings where advanced molecular testing is not routinely available. These models leverage routinely collected H&E-stained tissue slides, requiring no additional sample preparation or costly assays. Here we report HistoNav, a deep learning prognostic tool that exemplifies this approach, operating entirely on routine H&E-stained WSIs without requiring additional molecular testing. A detailed comparison of HistoNav against existing histopathology-based and molecular prognostic tools, together with its associated clinical and cost benefits, is presented in the Discussion, further supporting its value as a prognostic tool for early-stage CRC.

## 2. Materials and Methods

### 2.1. Study Design and Patient Cohorts

This retrospective study included 2095 patients with Stage II or IIIA CRC drawn from three cohorts: QUASAR 2 (*n* = 1176, of whom *n* = 1117 had evaluable DSS outcomes), used for model training and five-fold cross-validation; TCGA-COAD (*n* = 248, of whom *n* = 233 had evaluable DSS outcomes), used as an independent external validation cohort; and SCOT (*n* = 671, of whom *n* = 413 had evaluable DSS outcomes), held out entirely for independent external validation [20,21,22]. QUASAR 2 and SCOT were international, multicentre randomised controlled trials evaluating adjuvant chemotherapy regimens in CRC [20,21,23]; TCGA-COAD data were sourced from The Cancer Genome Atlas [22]. TCGA-READ (rectal adenocarcinoma) was not included in this study: TCGA had temporarily withdrawn whole-slide images from public access at the time of data curation to remove patient- and laboratory-identifying information from slide label regions, and only TCGA-COAD WSIs, obtained from data previously downloaded and stored by the authors, were available for curation. The SCOT cohort had no involvement at any stage of model development or threshold selection. A total of 1800 whole-slide images (WSIs) were used across the 1763 patients with evaluable DSS outcomes, as some patients contributed more than one WSI: 902 WSIs from 892 QUASAR2 patients were used for model training and five-fold cross-validation; the remaining 898 WSIs were reserved for validation, comprising 229 WSIs from the 225-patient QUASAR2 locked holdout test set, 238 WSIs from all 233 evaluable TCGA-COAD patients, and 431 WSIs from all 413 evaluable SCOT patients. Patient clinical characteristics are summarised in Table 1.

The primary endpoint was disease-specific survival (DSS), defined as time from surgical resection to cancer-specific death. Patients who died within 100 days of resection were excluded to minimise inclusion of deaths attributable to surgical complications. Patients with unavailable or incomplete DSS follow-up data were excluded entirely from model development and evaluation (rather than retained as right-censored observations); this accounts for the difference between total cohort size and the number of patients with evaluable DSS outcomes reported for TCGA-COAD and SCOT above. The study was conducted under data access agreements for the respective clinical trial datasets and the TCGA data repository; all data were used in de-identified form in accordance with the original ethics approvals of the source studies.

### 2.2. Histological Image Processing

Routine H&E-stained FFPE WSIs from surgically resected tumour specimens were digitised and processed to identify the tumour region of interest (ROI) via binary segmentation (lesion vs. normal tissue); the ROI collectively encompasses tumour epithelium, cancer-associated stroma, and tumour-infiltrating immune cells, rather than these being segmented as separate classes. ROI segmentation was performed using a supervised deep learning model trained on over 1100 pathologist-annotated QUASAR2 WSIs, based on an atrous-convolution semantic segmentation architecture [24]; stain normalisation was applied to all slides prior to downstream analysis. A Macenko-based colour normalisation algorithm [25] was used, with reference stain-vector parameters derived from QUASAR2 via a weighted-average procedure; normalisation was applied uniformly at both training and inference time, consistently across all three cohorts. Only pixels within the ROI were carried forward for feature extraction. ROI segmentation used a two-stage dual-model architecture: an initial model operated on the whole slide image at 5× magnification to generate a global candidate-region mask, and a second model operated at 20× magnification on tiles selected along the candidate region’s contour to refine the segmentation boundary at finer resolution (256 × 256-pixel tiles at 5× magnification for the initial model; for the refinement model, the corresponding 512 × 512-pixel field of view at 20× magnification is divided into 16 non-overlapping 128 × 128-pixel tiles); full architecture details are provided in the Supplementary Methods (Section S1). Full details of the segmentation architecture, training procedure, and normalisation parameters are provided in the Supplementary Methods (Section S1). WSIs were acquired using Leica Aperio AT2 scanners (Leica Biosystems Imaging, Inc., Vista, CA, USA) for QUASAR2 and SCOT (40× magnification, 0.253 µm/pixel) and, for TCGA-COAD, high-throughput Leica Aperio ScanScope scanners (40× magnification at 0.252, 0.2468, or 0.2456 µm/pixel, or 20× magnification at 0.2325 µm/pixel, depending on the individual slide); tile extraction (Section 2.3) rescales all tiles to a common physical field of view using QUASAR2’s resolution as reference (Supplementary Methods, Section S2).

### 2.3. AI Model Architecture

Morphological features were extracted from 256 × 256-pixel tiles at 40× magnification using a self-supervised Vision Transformer (ViT) trained under a DINO (self-distillation with no labels) framework [26,27], applied to all QUASAR2 WSIs without tile-level annotation. This approach enables the model to learn rich representations of tumour microenvironment architecture and cellular organisation directly from unlabelled histological data. Final model configuration comprised depth L = 12, six attention heads, and 384-dimensional feature embeddings; full hyperparameter selection is detailed in the Supplementary Methods (Section S2; Figure S1).

**Figure 1:**
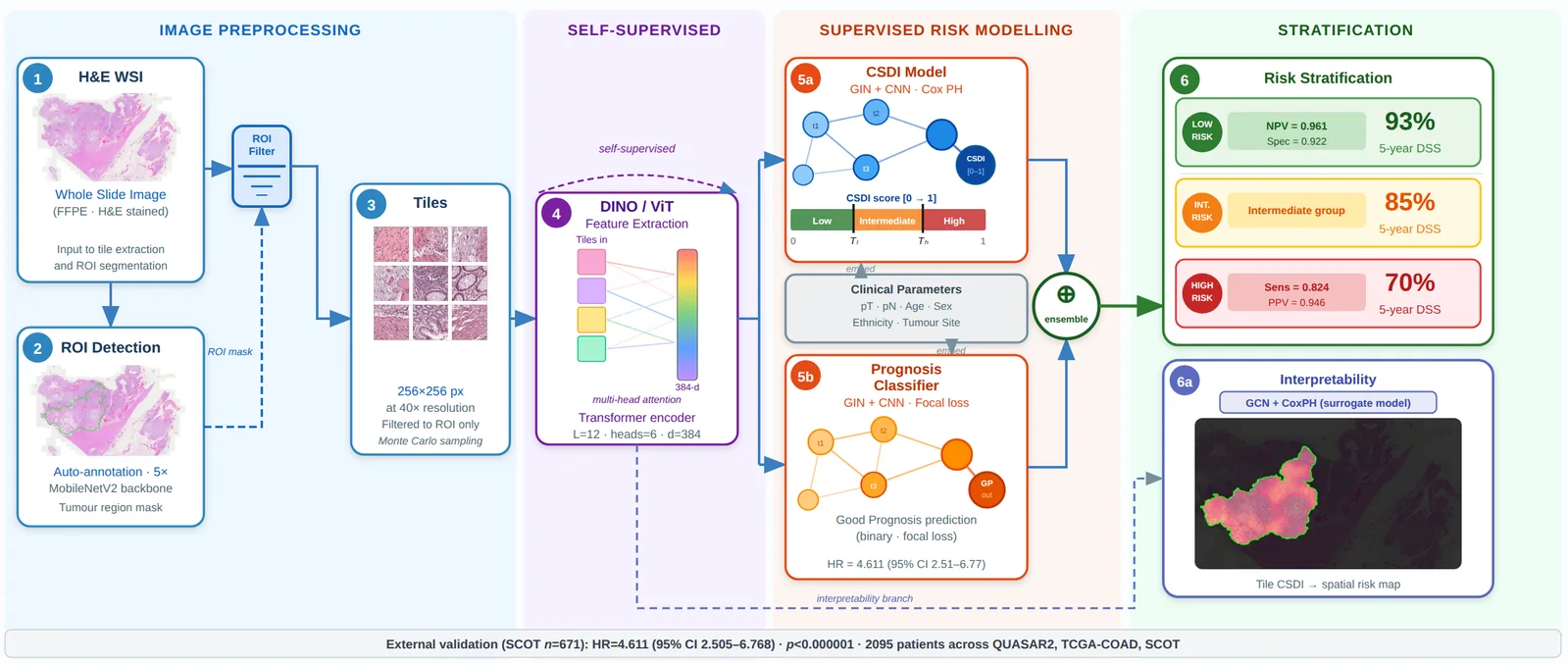
HistoNav CRC Prognostic Prediction Model Framework. Overview of the image processing and AI-based risk classification pipeline, from H&E-stained whole slide image input through ROI segmentation, DINO/ViT feature extraction, spatial graph construction, and GIN + CNN-based risk prediction to final three-tier patient stratification (low-, intermediate-, high-risk).

Tile features were used to construct a spatial graph for each WSI, with each tile as a node connected to its spatial neighbours within a defined distance threshold, preserving the topological organisation of the tumour microenvironment across the whole slide. Two parallel Graph Isomorphic Network (GIN) models with convolutional neural network (CNN) layers were trained on these graphs: (i) a Cancer-Specific Death Index (CSDI) model, derived from Cox proportional hazards regression [28], generating a continuous risk score bounded between 0 and 1; and (ii) a Good Prognosis Class Prediction model, a binary classifier identifying patients with favourable outcomes. Selected clinical variables (age, sex, pT stage, pN stage, tumour site) were incorporated as additional inputs to the CNN layer. The two models were combined in an ensemble framework to produce the final risk assignment. The three-tier low-/intermediate-/high-risk label is therefore a downstream, clinically motivated discretisation of these two model outputs—the continuous CSDI score and the binary Good Prognosis Class prediction—rather than a native three-class classification target. Full model architecture, loss functions (including a focal loss formulation to address class imbalance [29]), and training details are described in the Supplementary Methods (Sections S3–S6). Briefly, Stage 1 (the DINO/ViT encoder) was trained for 100 epochs using the AdamW optimiser (torch.optim.AdamW, PyTorch 2.0.1, version stated above, β = (0.9, 0.999); weight decay 5 × 10^−4^), with a linear learning-rate warm-up over the first 10 epochs to a peak of 5 × 10^−4^ followed by cosine annealing to 1 × 10^−6^, at a batch size of 64 per GPU; Stage 2 (the GIN + CNN risk-classification models) was trained for 100 epochs using the Adam optimiser (base learning rate 1 × 10^−3^; β = (0.9, 0.999); weight decay 0) at a batch size of 192. Both stages were trained on an IBM AC922 system (4× NVIDIA Tesla V100 GPUs, 32× IBM POWER9 CPU cores, 512 GB DDR4 RAM, RHEL 8) using Python 3.10, PyTorch 2.0.1, and CUDA 12.1 (Stage 2 additionally using the Deep Graph Library, DGL 2.2.1, for the GIN component). Full training hyperparameters are provided in Supplementary Methods Section S10. The overall image-processing and risk-classification pipeline is summarised in Figure 1.

### 2.4. Risk Classification and Interpretability

The CSDI and Good Prognosis Class outputs were combined to assign each patient to one of three prognostic tiers: low risk (good prognosis), intermediate risk, or high risk (poor prognosis). Classification thresholds were defined empirically on the QUASAR2 internal validation set based on the DSS endpoint and fixed prior to any external validation. The low- and high-risk thresholds were each selected using the pooled out-of-fold predictions from the five-fold cross-validation on the development dataset, as the value achieving a negative predictive value of at least 90% while maximising sensitivity, subject to allocating approximately 80% of patients to the low-risk group and approximately 10% to the high-risk group. They were not adjusted on the TCGA-COAD or SCOT cohorts (Supplementary Methods, Section S7).

To support pathologist review and clinical transparency, a Graph Convolutional Network (GCN) combined with Cox proportional hazards modelling, following established approaches for integrating deep neural networks with survival analysis [30], was trained in parallel to the primary GIN + CNN model on the same spatial tile graph. This surrogate model generates a CSDI for each individual tile, quantifying its local contribution to prognostic risk. These tile-level risk indices were visualised as colour heatmaps overlaid on the original WSI, enabling identification of the morphological regions most informative to each patient’s risk classification (Supplementary Methods, Section S8).

### 2.5. Statistical Analysis

Survival analyses were performed using the Kaplan–Meier estimator, with between-group comparisons by log-rank test. Univariate and multivariate Cox proportional hazards regression was used to assess the independent prognostic contribution of HistoNav risk classification alongside clinicopathological covariates (age, sex, pT stage, pN stage, MSI/MMR status); the proportional hazards assumption was verified using Schoenfeld residuals. Model performance was quantified for the high-risk and low-risk classifications separately, reporting sensitivity, specificity, positive predictive value (PPV), and negative predictive value (NPV) with 95% confidence intervals. For each risk-tier classification, we additionally report Risk-Tier Precision (RTP): the proportion of patients assigned to that tier by HistoNav who were correctly classified (equivalent to PPV for the High-Risk tier and to NPV for the Low-Risk tier). For internal model evaluation, approximately 20% of the QUASAR2 cohort (*n* = 225) was reserved as a locked holdout test set prior to any model development. A stratified sampling algorithm was applied to ensure proportional representation of each risk group: 20.64%, 19.78%, and 20.15% of the low-, high-, and intermediate-risk patients, respectively, were allocated to the test set, with the remaining 80% constituting the development dataset (full per-fold breakdown in Table S1; Supplementary Methods, Section S9). Five-fold cross-validation was then performed on the development dataset for hyperparameter selection and model evaluation. Risk classification thresholds used to define the low-, intermediate-, and high-risk groups (Supplementary Methods, Section S7) were likewise fixed using only the five-fold cross-validation evaluation folds from the development dataset; the *n* = 225 holdout test set was not used at any stage of threshold determination or model selection, and was reserved solely for final performance assessment (Supplementary Methods, Section S9). The TCGA-COAD and SCOT cohorts served as independent external validation sets, having played no role in model development. All analyses were performed in Python 3.

## 3. Results

### 3.1. Model Performance

The final HistoNav model—a GIN + CNN architecture with 40× tile resolution and 1/32 Monte Carlo tile subsampling—was evaluated on three datasets. Internal validation was performed on the locked QUASAR2 holdout test set (*n* = 225), which was reserved prior to any model development and played no role in training or hyperparameter optimisation. Independent external validation was performed on two additional cohorts: TCGA-COAD (*n* = 233 with evaluable DSS outcomes, from a total of 248 patients) and SCOT (*n* = 413 with evaluable DSS outcomes, from a total of 671 patients). HistoNav demonstrated strong generalisation across all three cohorts, together with interpretability through tile-level GCN-derived risk index heatmap overlays on whole slide images, designed to support pathologist review of the morphological regions contributing to each patient’s risk classification. This final 1/32 Monte Carlo subsampling ratio, selected during Stage 2 GIN + CNN training, is distinct from the 20% Monte Carlo tile sampling used during Stage 1 DINO/ViT self-supervised pretraining; see Supplementary Methods, Sections S2 and S10, for the full two-stage tile-sampling protocol.

**Figure 2:**
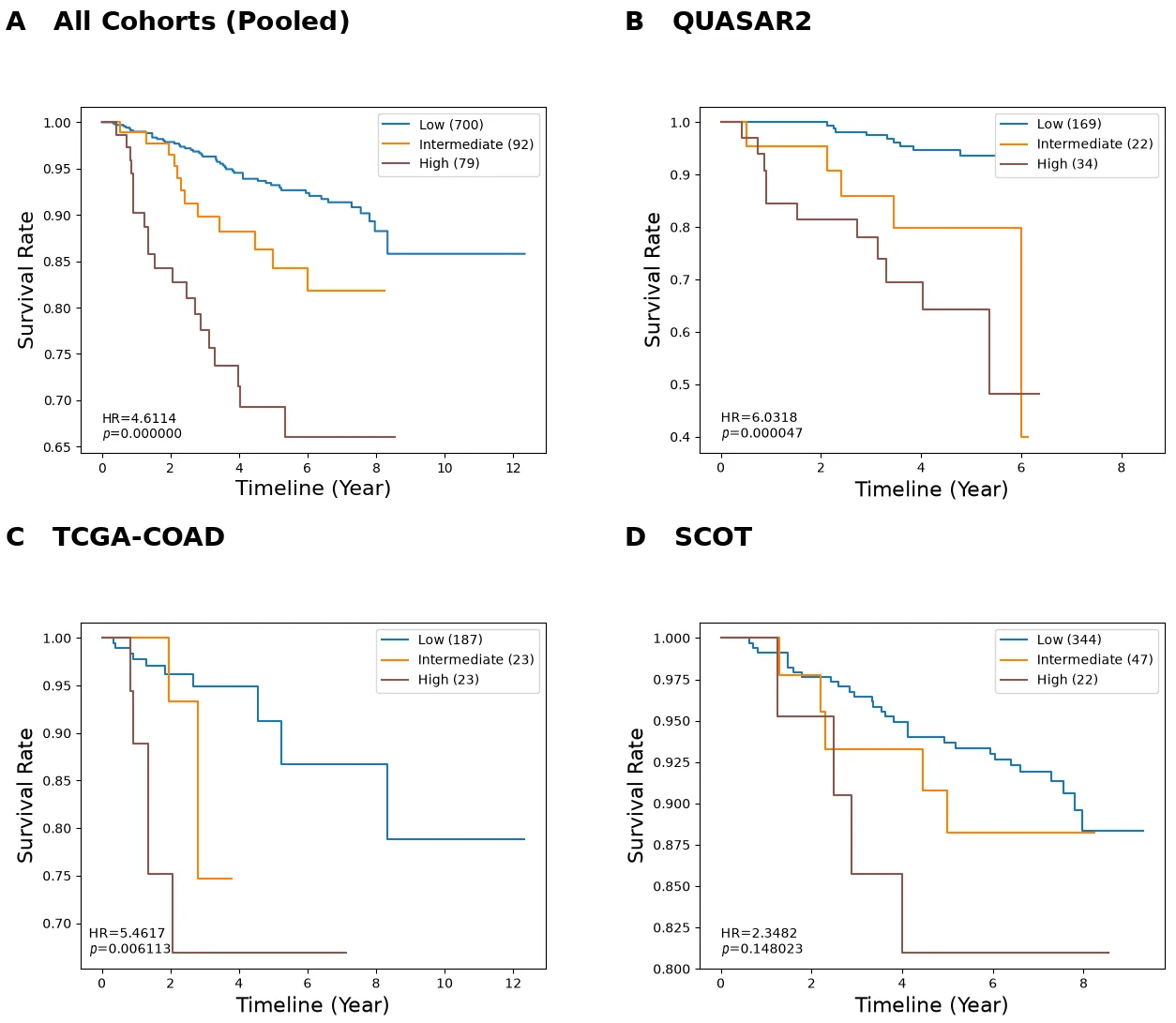
Kaplan–Meier Disease-Specific Survival Curves by HistoNav Risk Group. (**A**) Pooled across all three cohorts (*n* = 871: low-risk *n* = 700, intermediate-risk *n* = 92, high-risk *n* = 79); five-year DSS rates were 93.2% (low-risk), 84.3% (intermediate-risk), and 69.3% (high-risk), with HR (high vs. low) = 4.611 (95% CI: 2.776–7.662), log-rank *p* < 0.000001. Kaplan–Meier curves for disease-specific survival (DSS) stratified by HistoNav risk classification are also shown separately for each of the three cohorts: (**B**) QUASAR2 locked holdout test set (*n* = 225: low-risk *n* = 169, intermediate-risk *n* = 22, high-risk *n* = 34); five-year DSS 93.6% (95% CI: 87.9–96.6%) low-risk, 79.8% (95% CI: 54.2–92.0%) intermediate-risk, 64.2% (95% CI: 42.6–79.4%) high-risk, HR = 6.032 (95% CI: 2.729–13.330), log-rank *p* = 0.000047; (**C**) TCGA-COAD (*n* = 233: low-risk *n* = 187, intermediate-risk n = 23, high-risk *n* = 23); 91.3% (95% CI: 78.9–96.5%) low-risk, 74.7% (95% CI: 24.6–94.1%) intermediate-risk, 66.9% (95% CI: 36.8–85.0%) high-risk, HR = 5.462 (95% CI: 1.890–15.780), *p* = 0.006113; (**D**) SCOT (*n* = 413: low-risk *n* = 344, intermediate-risk *n* = 47, high-risk *n* = 22); 93.7% (95% CI: 90.5–95.8%) low-risk, 88.2% (95% CI: 73.9–94.9%) intermediate-risk, 81.0% (95% CI: 56.9–92.4%) high-risk, HR = 2.348 (95% CI: 0.834–6.614), *p* = 0.148023 (not statistically significant, reflecting SCOT’s smaller high-risk subgroup, *n* = 22).

As shown in Figure 2A, Kaplan–Meier analysis revealed a 5-year DSS of 93.2% (95% CI: 90.7–95.0%), 84.3% (95% CI: 73.1–91.1%), and 69.3% (95% CI: 55.7–79.4%) for the low-, intermediate-, and high-risk groups, respectively. The hazard ratio for the high versus low-risk group (HR = 4.611, 95% CI: 2.776–7.662; *p* < 0.000001) was statistically significant, demonstrating HistoNav’s potential to stratify patients by prognostic risk. The concordance index (C-index) for the final model, averaged across five-fold cross-validation on the QUASAR2 development dataset, was 0.649 (SD 0.037) on training folds and 0.592 (SD 0.039) on validation folds (Supplementary Methods, Section S10; full per-configuration C-index values in Figure S1). The area under the receiver operating characteristic curve (AUC) for low-risk classification was 0.72, consistent with published WSI-based prognostic models in colorectal cancer, where modest global discrimination reflects the inherent heterogeneity of H&E morphology. To assess robustness across AJCC stage subgroups and evaluate discrimination over time, Kaplan–Meier curves stratified by HistoNav risk group were generated separately for Stage II and Stage IIIA patients, and time-dependent (incident) AUC was estimated at 1, 3, and 5 years using inverse probability of censoring weighting; both analyses showed prognostic separation and discrimination consistent with the pooled results reported here (Supplementary Methods, Section S10; Figures S2 and S3). The clinical utility of HistoNav rests primarily on its high NPV (0.961), which supports confident identification of patients unlikely to benefit from adjuvant chemotherapy.

#### 3.1.1. Internal Validation

Classification performance metrics derived from Table 2 further highlight HistoNav’s clinical utility in identifying patients with a favourable prognosis. In the QUASAR2 internal test set, the model achieved a specificity of 0.883 and an NPV of 0.953, indicating that 95.3% of patients not classified as high-risk did not experience disease-specific death. Considered from the low-risk classification itself, 82.1% of true low-risk patients were correctly identified as such, and 94.7% of patients classified as low-risk truly had a favourable outcome. By contrast, the high-risk classification alone showed more modest precision: only 52.6% of true high-risk patients were correctly flagged as high-risk (sensitivity), and only 29.4% of patients classified as high-risk actually experienced disease-specific death (positive predictive value)—consistent with our recommendation that intermediate- or high-risk classifications prompt further diagnostic testing and clinical review rather than being acted on directly (Section 4). Accuracy across all three cohorts ranged from 0.794 to 0.891. These findings support HistoNav’s suitability as a decision-support tool, particularly for ruling out recurrence risk in the low-risk population.

#### 3.1.2. Independent External Validation

To examine whether HistoNav’s performance generalised across data sources with differing imaging protocols, staining characteristics and scanner hardware, Table 2 presents a per-cohort performance breakdown for the QUASAR2 internal test set (*n* = 225) and the two independent external validation cohorts: TCGA-COAD (*n* = 233) and SCOT (*n* = 413). The clinically critical metrics for ruling out disease-specific death were strikingly consistent across all three datasets. The negative predictive value for low-risk classification—the key metric underpinning the decision to safely withhold adjuvant chemotherapy—remained stable at 0.953 (QUASAR2), 0.967 (TCGA), and 0.954 (SCOT), with an overall NPV of 0.961 across all 871 evaluable patients. Specificity for low-risk classification showed similarly minimal variation (0.883, 0.919, and 0.943, respectively). By contrast, positive predictive value for the high-risk classification alone was more modest and variable across cohorts (0.294 QUASAR2, 0.217 TCGA, 0.161 SCOT; 0.190 pooled across all cohorts), reflecting the smaller and less consistently represented high-risk subgroup in each dataset. Considered directly, 94.6% of patients classified as low-risk across all three cohorts truly had a favourable outcome (Table 2, RTP row). Overall accuracy ranged narrowly from 0.853 to 0.893 across cohorts. High-risk sensitivity showed greater variability (0.526 in QUASAR2, 0.417 in TCGA, and 0.269 in SCOT), reflecting differences in the proportion and absolute number of high-risk events across cohorts as well as the inherent difficulty of classifying the most extreme risk cases in externally sourced datasets. Collectively, the consistency of NPV and specificity across three independent cohorts acquired on different scanner platforms and with different tissue preparation protocols provides empirical evidence against image-based data variability as a material confounding factor in HistoNav’s prognostic performance.

### 3.2. Univariate Analysis

Univariate Cox proportional hazards analysis was performed to identify clinical and pathological factors independently associated with disease-specific survival (Table 3). pT stage (HR = 2.731, 95% CI: 1.865–3.999; *p* < 0.001) and pN stage (HR = 3.404, 95% CI: 1.845–6.278; *p* < 0.001) were the strongest prognostic factors. Ethnicity was also significantly associated with disease-specific survival (HR = 3.077, 95% CI: 1.909–4.960; *p* < 0.001), as was tumour site (HR = 1.770, 95% CI: 1.171–2.676; *p* = 0.0067). In contrast, sex (HR = 0.996, 95% CI: 0.549–1.809; *p* = 0.990) and age (HR = 0.984, 95% CI: 0.955–1.014; *p* = 0.289) were not statistically significant. MSI/MMR status was not independently prognostic (HR = 1.190, 95% CI: 0.740–1.920; *p* = 0.465; *n* = 772), consistent with its established role as a predictive rather than purely prognostic biomarker in this population.

### 3.3. Multivariate Analysis

To identify independent prognostic factors, multivariate Cox regression analysis was performed incorporating all variables identified in univariate analysis, alongside the HistoNav risk classification (Table 4). pT stage remained an independent predictor of disease-specific survival after adjustment (HR = 1.987, 95% CI: 1.241–3.182; *p* = 0.0042). pN stage also retained independent significance (HR = 2.057, 95% CI: 1.079–3.919; *p* = 0.0283). Ethnicity (HR = 1.490, 95% CI: 0.874–2.063; *p* = 0.178) and tumour site (HR = 1.342, 95% CI: 0.740–3.000; *p* = 0.264) did not retain independent prognostic significance after adjustment for other covariates. HistoNav risk classification was confirmed as an independent prognostic factor, demonstrating that the model’s predictive value is not confounded by established clinicopathological variables and provides additional prognostic information beyond conventional staging.

## 4. Discussion

This study presents HistoNav, an AI-based prognostic stratification tool that analyses routine haematoxylin and eosin (H&E)-stained whole slide images to classify Stage II/IIIA CRC patients into low-, intermediate-, and high-risk groups for disease-specific survival. The model demonstrated a 4.6-fold difference in 5-year DSS between the high- and low-risk groups (HR = 4.611, 95% CI: 2.776–7.662; *p* < 0.000001), confirming robust and clinically meaningful prognostic discrimination. Independent validation in the SCOT cohort demonstrated generalised applicability, supporting the external validity of the model.

The most clinically significant performance characteristic of HistoNav is its high NPV of 0.961 for the low-risk classification. This indicates that 96.1% of patients designated as low-risk by the model will not experience a cancer-specific death—a property of direct relevance to adjuvant chemotherapy decision-making. Adjuvant chemotherapy is administered to over 50% of Stage II/IIIA CRC patients following curative resection, yet the majority of this group has an inherently favourable prognosis and is unlikely to benefit from systemic treatment [20,21]. The MOSAIC trial, which established FOLFOX4 as adjuvant therapy for colon cancer, reported improved 6-year overall survival in Stage III patients (HR 0.80, *p* = 0.023) but found no significant OS benefit in the Stage II subgroup [31], underscoring the need for risk-stratified selection. The toxicity burden of adjuvant fluoropyrimidine- or oxaliplatin-based regimens is well established, and the ability to identify patients in whom treatment can be safely withheld represents a meaningful advance in personalised oncology. The high specificity (0.922) of HistoNav for the low-risk class further supports its utility as a tool for ruling out recurrence risk, rather than merely flagging it.

The emergence of ctDNA as a postoperative biomarker for minimal residual disease has significantly advanced personalised treatment selection in early-stage CRC. The DYNAMIC randomised trial demonstrated that a ctDNA-guided approach to adjuvant chemotherapy in Stage II colon cancer reduced chemotherapy use without compromising two-year recurrence-free survival, with five-year follow-up confirming sustained benefit [32,33]. Similarly, the CIRCULATE-Japan GALAXY study established that ctDNA-based molecular residual disease detection is strongly associated with recurrence risk and benefit from adjuvant chemotherapy in resectable CRC [34]. HistoNav and ctDNA-based approaches are likely to be complementary rather than competing tools. HistoNav operates at the time of surgical resection using tissue already collected during routine pathology processing, without additional sampling, laboratory infrastructure, or turnaround time. ctDNA testing, by contrast, requires a postoperative blood draw at a defined timepoint, is subject to analytical sensitivity limitations in patients with low tumour burden, and adds cost to the care pathway [35]. A sequential decision framework in which HistoNav first identifies the low-risk population who may safely forgo both adjuvant chemotherapy and ctDNA surveillance, while ctDNA testing is reserved for intermediate- and high-risk patients to further refine treatment intensity, represents an attractive and resource-efficient clinical model that warrants prospective evaluation.

Several histopathology image-based AI prognostic models have been reported in CRC and other solid tumours [10,11,12]. Sun et al. demonstrated that deep learning applied to WSIs can improve prognostic risk stratification in Stage III CRC beyond TNM staging alone [36]. More recently, Zhao et al. developed SurvFinder, a deep learning framework identifying tertiary lymphoid structures (TLS) as prognostic biomarkers in Stage II CRC across four Chinese cohorts (*n* = 1604; HR 8.23 for high versus low risk), though—as the authors note—without prospective validation or real-world clinical deployment [37]. HistoNav extends this paradigm to the earlier, clinically more challenging Stage II/IIIA population, where individual prognosis is particularly difficult to determine using conventional clinicopathological criteria. Compared with existing molecular prognostic signatures—such as gene expression profiles (e.g., ColoPrint [38,39], Oncotype DX Colon [40]) or immunohistochemical markers (e.g., CDX2 [41,42])—HistoNav operates entirely on digitised H&E slides, requiring no additional tissue preparation, molecular assays, or proprietary reagents and outperforms these tests in terms of hazard ratios, lending greater evidential weight to this test. This characteristic makes it both cost-effective and scalable across healthcare systems with varying levels of molecular testing infrastructure. The independent prognostic value confirmed by multivariate analysis, where HistoNav risk classification remained significant after adjusting for pT and pN stage, further distinguishes the model from a simple algorithmic reflection of conventional staging.

Among the growing body of H&E image-based AI prognostic tools for early-stage CRC, comparators were selected using three criteria: (i) study populations restricted to, or inclusive of, Stage II and/or IIIA colorectal cancer; (ii) disease-specific survival, or the closely equivalent cancer-specific survival, reported as a primary endpoint; and (iii) peer-reviewed publication within the last six years (2020–2026). Two comparators are retained as deliberate exceptions to these criteria: OncoProg [43] falls outside the six-year recency window but is included as the closest available historical precedent for a three-tier DSS-based stratification and the only comparator with a published cost-effectiveness analysis; QuantCRC [44] reports recurrence-free survival rather than disease-specific survival as its primary endpoint but is included because it shares HistoNav’s H&E-only input modality. Applying these criteria, three tools bear closest comparison to HistoNav. Skrede et al. at Oslo University Hospital developed Histotyping, a deep learning classifier trained on H&E-stained WSIs from four cohorts, which predicted cancer-specific survival with a hazard ratio of 3.84 for poor versus good prognosis in an independent external test set [45]. OncoProg (formerly ColoProg), developed by Danielsen et al., takes a complementary image-based approach: digital image analysis of routine H&E slides is used to quantify DNA ploidy and stroma-tumour fraction, generating a three-tier prognostic score validated in approximately 1000 Stage II CRC patients across three cohorts including QUASAR 2 [43]. That analysis reported 5-year cancer-specific survival of 90%, 83%, and 73% for low-, intermediate-, and high-risk groups, respectively—closely paralleling HistoNav’s 93%, 84%, and 69% DSS. A third H&E-based comparator, QuantCRC, applies a deep learning algorithm to extract 15 quantitative histopathologic features—including stromal subtype, tumour grade, tumour budding, and tumour-infiltrating lymphocytes—from routine H&E slides, stratifying Stage II/III CRC patients into low-, intermediate-, and high-risk groups for recurrence-free survival; in an external validation cohort, 36-month recurrence rates were 32.7% versus 13.4% for high- versus low-risk Stage III patients, and 15.8% versus 5.4% for high- versus low-risk Stage II patients [44]. Unlike HistoNav’s end-to-end self-supervised representation learning, QuantCRC relies on a fixed, hand-curated feature set derived from pathologist-informed morphological categories, rather than features learned directly from the data. HistoNav advances beyond these approaches through its self-supervised Vision Transformer (DINO/ViT) feature extraction and graph isomorphic network spatial modelling of tile relationships across the tumour microenvironment, capturing tissue architecture at a representational depth not accessible to classical morphometry or shallower CNN architectures (see Table 5 for Comparison of HistoNav with established prognostic biomarkers).

This study has several limitations that should be acknowledged. The retrospective design, while based on data from large, well-characterised international randomised controlled trials (QUASAR2, SCOT) and a major public genomic atlas (TCGA), limits causal inference and may be subject to selection biases inherent to the source cohorts. Rectal cancer cases were present only in the QUASAR2 training/internal-validation cohort (*n* = 158) and were not represented in either external validation cohort; the generalisability of HistoNav’s performance to rectal cancer specifically has therefore not yet been demonstrated in an independent dataset; this reflects a data-availability constraint (Methods, Section 2.1) rather than a deliberate scope decision. By design, a HistoNav low-risk classification is intended to support withholding adjuvant chemotherapy; a high-risk classification was originally intended to support consideration of combination (multi-agent) adjuvant chemotherapy, and an intermediate-risk classification to support consideration of single-agent adjuvant chemotherapy. Given the current model’s more modest positive predictive value at the high-risk extreme (Table 2; pooled positive predictive value 0.190 across all cohorts), we consider the present version of HistoNav validated primarily for confidently identifying low-risk patients suitable for chemotherapy de-escalation; patients classified as intermediate- or high-risk should undergo further diagnostic testing and clinical review, rather than treatment intensity being determined by the HistoNav classification alone. The greater cross-cohort variability in high-risk sensitivity (52.6% in QUASAR2, 41.7% in TCGA-COAD, and 26.9% in SCOT; Table 2) is a more direct signature of sensitivity to domain shift than the low-risk NPV and specificity metrics discussed above, which remained comparatively stable across cohorts; we interpret this primarily as reflecting the small and heterogeneously represented high-risk subgroup in each external cohort, though we cannot exclude a contribution from between-cohort differences in scanner platform, staining protocol, or case mix [46] (Supplementary Methods, Section S11.1). This pattern reinforces our recommendation that HistoNav’s current validated use be scoped to confident low-risk identification, where performance is most consistent, rather than to high-risk classification, where cross-cohort variability is greatest. The intermediate-risk group (5-year DSS 85%) presents a clinical challenge: this stratum does not clearly benefit from withholding or administering adjuvant chemotherapy based on HistoNav alone, and additional biomarkers—including ctDNA, MMR/MSI status, or molecular profiling—may be required to guide decisions in this population. Some covariates, including ethnicity and tumour site, had incomplete data in the multivariate analysis, which may limit the generalisability of the multivariate findings. Comparisons with existing histopathology-based and molecular prognostic tools (Table 5) are literature-based, drawing on each tool’s own reported performance in its own validation cohort, rather than a controlled head-to-head benchmark on a shared test set (see also Supplementary Methods, Section S11.2); the trained models or implementations of these external tools were not available to us for direct comparison. Finally, prospective, interventional clinical validation is required before HistoNav can be integrated into clinical practice; for example, postoperative ctDNA testing in a sequential decision framework could be explored in a randomised or prospective cohort design. Formal qualitative and quantitative pathologist-concordance evaluation of the GCN-derived interpretability heatmaps has not yet been performed; this is planned as part of the prospective clinical utility study. HistoNav’s self-supervised DINO/ViT encoder was developed and trained from scratch on QUASAR2 before the emergence of mature, large-scale public histopathology foundation models such as UNI [15]. A preliminary, small-scale comparison with more recent self-supervised vision transformer variants (DINOv2, DINOv3) did not show a clear improvement over the original DINO configuration used in this study; this comparison was not conducted systematically, given the substantial computational cost of full model retraining, and a more rigorous benchmark against these and other publicly available histopathology foundation models is planned as future work. Extension of the model to Stage I and Stage IIIB/C CRC, as well as other tumour types where H&E-based prognostic discrimination is clinically relevant, are natural next steps. Table 5 summarises the key performance and practical characteristics of HistoNav relative to these established histopathological and molecular prognostic approaches.

## 5. Conclusions

HistoNav demonstrates that deep learning applied to routine H&E-stained WSIs can deliver clinically meaningful prognostic stratification for Stage II/IIIA CRC without requiring additional laboratory testing, molecular assays, or specialist infrastructure beyond standard digital pathology. Validated across 2095 patients from three independent cohorts with differing imaging protocols and tissue preparation workflows, the model achieved a 4.6-fold difference in disease-specific survival between high- and low-risk groups (HR = 4.611, 95% CI: 2.776–7.662; *p* < 0.000001), with five-year DSS rates of 93.2% (95% CI: 90.7–95.0%), 84.3% (95% CI: 73.1–91.1%), and 69.3% (95% CI: 55.7–79.4%) for low-, intermediate-, and high-risk patients respectively. The consistency of key performance metrics across QUASAR2, TCGA-COAD, and SCOT provides robust evidence of generalisation across clinical and technical heterogeneity.

## 6. Patents

The authors have filed a patent application relevant to the technology described in this manuscript: UK Patent Application No. 2406267.1, “Histopathology-based solid tumour analysis” (published 5 November 2025 as GB 2640747).

## Figures and Tables

**Table 1:** Patient Clinical Data Characteristics.

	QUASAR2	TCGA	SCOT	Total
**Gender**				
Male	643	122	257	1022
Female	474	111	156	741
**Age** (Years)				
Min	27	31	24	24
Max	85	85	83	85
Mean	63.9	64.8	62.4	63.7
Standard Deviation	9.6	12.6	9.4	10.0
**Ethnicity**				
White	1091	109	0	1200
Black	10	39	0	49
Others	8	11	0	19
Unknown	8	74	413	495
**AJCC Stage**				
II	408	145	267	820
IIIA	709	88	146	943
**pT**				
1	22	1	35	58
2	76	8	111	195
3	580	201	144	925
4	391	23	123	537
x	48	0	0	48
**pN**				
0	397	133	267	797
1	500	66	138	704
2	180	34	8	222
x	40	0	0	40
**Tumour Site**				
Left side colon	438	69	263	770
Right side colon	450	113	149	712
Rectum	158	0	0	158
Unknown	71	51	1	123

**Table 2:** Performance Matrix.

	QUASAR 2 Test Set (*n* = 225)		TCGA (*n* = 233)		SCOT (*n* = 413)		All Cohorts (*n* = 871)	
	High Risk	Low Risk	High Risk	Low Risk	High Risk	Low Risk	High Risk	Low Risk
TPR	0.526	0.700	0.417	0.385	0.269	0.048	0.326	0.441
TNR	0.883	0.821	0.919	0.814	0.943	0.880	0.922	0.824
PPV	0.294	0.375	0.217	0.109	0.161	0.022	0.190	0.175
NPV	0.953	0.947	0.967	0.957	0.954	0.943	0.961	0.946
RTP	0.294	0.947	0.217	0.957	0.161	0.943	0.190	0.946
ACC	0.853	0.804	0.893	0.790	0.879	0.836	0.891	0.794

For the High-Risk column, a positive result denotes a HistoNav high-risk classification; for the Low-Risk column, a positive result denotes a HistoNav non-low-risk (i.e., intermediate- or high-risk) classification. Both columns therefore use the convention that a positive result flags a patient as requiring closer clinical attention, with ground-truth positive defined as disease-specific death. Table 2 does not report per-class TPR/TNR/PPV/NPV for the intermediate-risk group, since this tier is a residual “neither low nor high” category with no associated HistoNav-directed treatment decision of its own (low-risk supports withholding chemotherapy and high-risk supports combination chemotherapy; intermediate-risk patients are managed per standard of care rather than a HistoNav-specific recommendation), so a defined “positive class” tied to a clinical decision threshold does not naturally apply. For descriptive completeness, the intermediate-risk group’s 5-year DSS was 85% (*n* = 92 pooled across cohorts; Figure 2). The Risk-Tier Precision (RTP) row restates, for each column, the precision of predicting that specific risk tier directly—i.e., of patients HistoNav classifies into this tier, the proportion for whom that classification was correct. For the High-Risk column, RTP equals the PPV row above; for the Low-Risk column, RTP equals the NPV row above, since under the positive-class convention defined above (positive = non-low-risk), NPV of that test is definitionally identical to the precision of predicting survival directly. RTP is provided for readers who prefer risk-tier-direct interpretation over the positive-class convention used elsewhere in this table. Abbreviations: TPR, true positive rate (sensitivity); TNR, true negative rate (specificity); PPV, positive predictive value; NPV, negative predictive value; RTP, risk-tier precision; ACC, accuracy.

**Table 3:** Univariate Analysis Results.

Parameter	Value	Hazard Ratio (95% CI)	*p*-Value
Gender	Female = 0, Male = 1	0.996 (0.549–1.809)	0.9902
Age	Years	0.984 (0.955–1.014)	0.2889
Ethnicity	0 = white, 1 = black, 2 = others	3.077 (1.909–4.960)	0.000004
pT	1–4	2.731 (1.865–3.999)	0.0000002
pN	0–2	3.404 (1.845–6.278)	0.000088
MSI/MMR	MSS = 0, MSI-H = 1	1.190 (0.740–1.920)	0.465
Tumour Site	0 = left, 1 = right, 2 = rectum	1.770 (1.171–2.676)	0.0067

**Table 4:** Multivariate Analysis Results.

Parameter	Value	Hazard Ratio (95% CI)	*p*-Value
Ethnicity	0 = white, 1 = black, 2 = others	1.490 (0.874–2.063)	0.1784
pT	1–4	1.987 (1.241–3.182)	0.0042
pN	0–2	2.057 (1.079–3.919)	0.0283
Tumour Site	0 = left, 1 = right, 2 = rectum	1.342 (0.740–3.000)	0.2639

**Table 5:** Comparison of HistoNav with established prognostic biomarkers for Stage II/IIIA colorectal cancer.

Characteristic	HistoNav	ctDNA (Liquid Biopsy)	ColoPrint	Oncotype DX Colon	CDX2	QuantCRC
**Technology/** **platform**	Deep learning on H&E WSI (DINO/ViT + GIN/CNN)	Circulating tumour DNA (next-generation sequencing)	18-gene expression microarray	12-gene RT-PCR recurrence score	Immunohistochemistry (IHC)	Deep learning quantitative histomorphometry on H&E WSI (15 features: stromal subtype, tumour grade, budding, TILs, etc.) [44]
**Specimen type**	Routine FFPE H&E section	Peripheral blood	Tumour RNA (fresh-frozen preferred)	FFPE tumour RNA	FFPE tumour section	Routine FFPE H&E section
**Sampling time** **relative to surgery**	At time of resection	4–7 weeks post-resection	At time of resection	At time of resection	At time of resection	At time of resection
**Additional laboratory infrastructure**	Digital pathology scanner (increasingly standard)	Specialist liquid biopsy laboratory	Microarray platform; centralised laboratory	Centralised RT-PCR facility (single vendor)	Standard IHC laboratory	Digital pathology scanner (same modality as HistoNav); commercial platform licence (Aiforia)
**Incremental cost**	Negligible (existing slides reused)	High	High; limited availability	High; centralised testing only	Low–moderate	Negligible slide cost; commercial licensing fee
**Key prognostic/clinical metric**	NPV 0.961; HR 4.61 (high vs. low risk; *p* < 0.000001) [this study]	ctDNA-guided ACT: ~50% reduction in chemotherapy use without compromising RFS [32,33]	Identifies ~60–73% of patients as low-risk [38,39]	Continuous recurrence score; validated in QUASAR trial (~1851 patients) [40]	CDX2 loss in ~5% of patients; confers high recurrence risk [41,42]	3-tier risk groups; 36-month recurrence 32.7% (high) vs. 13.4% (low), Stage III; 15.8% vs. 5.4%, Stage II [44]
**Independent** **validation cohort(s)**	2095 patients across 3 independent cohorts (QUASAR2, TCGA-COAD, SCOT)	DYNAMIC trial (*n* = 455) [32,33]; GALAXY study (*n* = 1074) [34]	Multiple cohorts (500–1000 patients per cohort) [38,39]	QUASAR trial (~1851 patients) [40]	Multiple cohorts [41,42]	6468 H&E slides (development *n* = 1928; internal test *n* = 483; external validation *n* = 938) [44]
**Randomised predictive evidence** **(ACT benefit)**	Prospective trial pending	Yes (DYNAMIC randomised controlled trial) [32,33]	No	No	No	No (prognostic only; currently Research Use Only/Performance Studies Only)

## Data Availability

The data supporting the findings of this study are available from the corresponding author upon reasonable request.

## Supplementary Materials

The following supporting information can be downloaded at: https://www.mdpi.com/article/10.3390/cancers18172863/s1, Supplementary Methods: Technical details of model architecture, training, and validation procedures, comprising Sections S1–S10 (Section S1: Tumour Region of Interest (ROI) Segmentation; Section S2: Self-Supervised Feature Extraction (DINO/ViT); Section S3: Spatial Graph Construction; Section S4: GIN + CNN Neural Network Architecture; Section S5: Cancer-Specific Death Index (CSDI) Model; Section S6: Good Prognosis Class Prediction Model; Section S7: Final Risk Classification and Threshold Determination; Section S8: GCN Surrogate Model for Spatial Interpretability; Section S9: Cross-Validation and External Validation Protocol; Section S10: Model Training Results); Section S11. Extended Discussion: Additional Robustness and Comparator Considerations; Table S1: Per-fold cross-validation and holdout risk-group allocation percentages; Figure S1: ViT Hyperparameter Optimisation for the DINO Self-Supervised Feature Extractor. Figure S2: Kaplan–Meier Disease-Specific Survival Curves by HistoNav Risk Group, Stratified by AJCC Stage; Figure S3: Time-Dependent (Incident) Receiver Operating Characteristic Curves at 1, 3, and 5 Years.

## Abbreviations

The following abbreviations are used in this manuscript:

ACC	Accuracy
AI	Artificial Intelligence
AJCC	American Joint Committee on Cancer
AUC	Area Under the (Receiver Operating Characteristic) Curve
CI	Confidence Interval
CNN	Convolutional Neural Network
CRC	Colorectal Cancer
CSDI	Cancer-Specific Death Index
CTC	Circulating Tumour Cell
ctDNA	Circulating Tumour DNA
DFS	Disease-Free Survival
DINO	Self-DIstillation with NO labels
DSS	Disease-Specific Survival
FFPE	Formalin-Fixed Paraffin-Embedded
GCN	Graph Convolutional Network
GIN	Graph Isomorphic Network
GNN	Graph Neural Network
H&E	Haematoxylin and Eosin
HR	Hazard Ratio
IHC	Immunohistochemistry
MMR	Mismatch Repair
MRD	Minimal Residual Disease
MSI	Microsatellite Instability
NPV	Negative Predictive Value
OS	Overall Survival
PPV	Positive Predictive Value
pN	pathological Node stage
pT	pathological Tumour stage
ROI	Region of Interest
RTP	Risk-Tier Precision
RT-PCR	Reverse Transcription Polymerase Chain Reaction
TCGA	The Cancer Genome Atlas
TME	Tumour Microenvironment
TNM	Tumour, Node, Metastasis
TNR	True Negative Rate (Specificity)
TPR	True Positive Rate (Sensitivity)
ViT	Vision Transformer
WSI	Whole Slide Image
