# Supplementary material for: HistoNav: AI-Based H&E Histopathology Predicts Disease-Specific Survival and Guides Adjuvant Chemotherapy Decisions in Stage II/IIIA Colorectal Cancer
Source: Cancers (Basel). 2026 Sep 4;18(17):2863. doi: 10.3390/cancers18172863 (PMC13564889; doi:10.3390/cancers18172863)
Supplement: Supplementary file 1 [file cancers-18-02863-s001.zip › cancers-4474635-supplementary.pdf]

HistoNav: Supplementary Methods

Technical details of model architecture, training, and validation procedures

Section S1. Tumour Region of Interest (ROI) Segmentation

WSI Acquisition and Scanning

QUASAR2 and SCOT whole slide images were digitised using Leica Aperio AT2 scanners at 40× magnification (0.253 µm/pixel, mpp). TCGA-COAD whole slide images, sourced from the TCGA data portal, were digitised using high-throughput Leica Aperio ScanScope scanners; native resolution varied by slide, comprising 40× magnification at 0.252, 0.2468, or 0.2456 µm/pixel, or 20× magnification at 0.2325 µm/pixel. This scanner and resolution heterogeneity for TCGA-COAD reflects the multi-institutional, retrospective nature of the TCGA archive rather than a controlled acquisition protocol. To ensure that every tile used for downstream feature extraction covers an equivalent physical field of view (FOV) regardless of source scanner, tile pixel dimensions were adjusted per native resolution before rescaling to a common reference size; see Section S2 (Tile Extraction) for the full procedure and per-resolution tile dimensions. Of the 238 TCGA-COAD whole slide images, 83 were natively digitised at 20× magnification (0.2325 µm/pixel) rather than 40×. As described in Section S2 (Tile Extraction), tiles extracted from these 20×-native slides are rescaled so that their physical field of view matches that of native 40× tiles from the other cohorts/scanners. Consequently, throughout this manuscript and its Supplementary Materials, magnification labels (e.g., 40×, 20×, 10×, 5×) denote the effective, FOV-normalised resolution tier used at each stage of the pipeline, rather than necessarily the literal native optical magnification reported by the source scanner for every individual slide: 40× denotes the reference (highest) resolution used throughout, and each successive label denotes a further twofold reduction (20× = downsampled ×2 relative to 40×, 10× = ×4, 5× = ×8).

The ROI segmentation model was based on the DeepLabv3+ architecture [24], a deep convolutional neural network (DCNN) that incorporates atrous spatial pyramid pooling (ASPP) to capture multi-scale contextual information by aggregating features at different receptive fields, enabling accurate delineation of complex tissue structures in high-resolution WSIs.

MobileNetV2 was used as the backbone for feature extraction. Its depth-wise separable convolutions and inverted residual structure provide computational efficiency without sacrificing representational capacity, making it well-suited for processing very large histopathology images.

Dual-Model Architecture

Two DeepLabv3+ instances were deployed in series to improve segmentation accuracy across scales:

- Model 1 receives the 5× magnification whole slide image and generates a global foreground mask identifying candidate tumour regions.
- Along the contour of the candidate region identified by Model 1, 256×256-pixel tiles at 5× magnification are selected for fine-grained refinement.
- For each 5×-magnification, 256×256-pixel tile selected along the candidate-region contour, the corresponding physical field of view is re-extracted at 20× magnification: since 20× represents 4× the magnification of 5×, this FOV corresponds to a 512×512-pixel image at 20× (equivalent to a 2×2 grid of four 256×256-pixel tiles). Model 2 divides this 512×512-pixel, 20×-magnification image into 16 non-overlapping, equal-size 128×128-pixel tiles, each of which is classified by Model 2 as ROI or non-ROI to refine the segmentation boundary.

Stain Normalisation

Prior to segmentation and all downstream processing, a Macenko-based H&E normalisation algorithm [25] was applied to correct for inter-slide colour variation arising from differences in staining protocols and scanner settings. This normalisation was applied uniformly at both training and inference time, to every slide processed by the pipeline. Normalisation reference parameters (mean and standard deviation of stain vectors) were derived from QUASAR2 images using a weighted average procedure. This normalisation was applied consistently across all three cohorts (QUASAR2, TCGA-COAD, SCOT).

Training Data and Augmentation

Over 1,100 H&E-stained WSIs from QUASAR2 with pathologist-annotated tumour boundaries were used for supervised training. Ground truth masks distinguished lesion tissue from normal tissue. The following augmentation strategies were applied during training to improve robustness to image variability:

- Random horizontal and vertical flipping
- Random resizing and rotation
- H&E stain augmentation (random perturbation of stain concentration)

Only pixels classified as ROI by both segmentation models were passed to the feature extraction stage.

Section S2. Self-Supervised Feature Extraction (DINO/ViT)

Morphological features were extracted using a self-supervised learning framework based on DINO (Self-DIstillation with NO Labels) [26], using a Vision Transformer (ViT) [27] as the

encoder backbone. This approach eliminates the need for tile-level annotations by learning discriminative representations from the structure of the data itself.

Student–Teacher Architecture

The DINO framework employs two encoder networks with identical architecture — a student network and a teacher network — trained simultaneously. Both networks receive the same input image transformed by different random augmentations. The teacher network output is centred using an exponential moving average (EMA) computed over the training batch, which prevents representation collapse without requiring contrastive pairs. Each network produces an N-dimensional feature vector normalised via temperature softmax over the feature dimension. Only the teacher encoder is saved and used at inference.

Tile Extraction

WSI tiles were extracted at 40× magnification as 256×256-pixel non-overlapping patches from within the ROI, with QUASAR2's native resolution (0.253 μm/pixel, Leica Aperio AT2) used as the reference physical field of view (FOV). Native tile pixel dimensions were scaled to match this reference FOV across cohorts/scanners with differing microns-per-pixel (mpp) resolution: 256×256 px at 0.253 mpp (QUASAR2, SCOT; Leica Aperio AT2); 257×257 px at 0.252 mpp; 263×263 px at 0.2468 mpp; 264×264 px at 0.2456 mpp; and 279×279 px at 0.2325 mpp (TCGA-COAD, per-slide, depending on native scanner resolution; Leica Aperio ScanScope). Tiles larger than 256×256 px were downsampled to 256×256 px, so that every tile used for feature extraction covers an equivalent physical tissue area regardless of source scanner. To reduce training time while maintaining representative coverage of each slide, a Monte Carlo random sampling strategy was used to select 20% of tiles per WSI, stratified to preserve the spatial distribution of tissue types across the slide.

Hyperparameter Selection

Extensive grid search was conducted over the following hyperparameters:

- Number of attention heads: 6, 8, 12
- Network depth (transformer layers): 6, 8, 12
- Attention drop rate: 0.0, 0.1, 0.2
- Drop-path rate: 0.0, 0.1, 0.2
- Gradient clipping value: 1.0, 3.0, 5.0
- Learning rate and optimiser schedule

Final selected configuration:

- Depth (L): 12 transformer layers
- Attention heads: 6
- Feature dimension: 384

- Gradient clipping: 3.0
- Drop-path rate: 0.1

Training Procedure

The DINO/ViT model was trained on all QUASAR2 WSIs. Training steps were as follows:

- Apply ROI segmentation (S1) to each WSI to define the analysis region.
- Extract 256×256-pixel tiles at 40× magnification from within the ROI.
- Apply Monte Carlo tile sampling (20% per slide).
- Feed sampled tiles to the student and teacher networks with different random augmentations.
- Optimise student parameters via the DINO self-distillation objective; update teacher parameters via EMA.
- After training, save the teacher encoder weights. Run all tiles in inference mode through the teacher encoder to extract 384-dimensional feature embeddings for each tile.

Section S3. Spatial Graph Construction

For each WSI, a spatial graph G = (V, E) was constructed to represent the topological organisation of the tumour microenvironment, where each tile within the ROI forms a node V and edges E encode spatial adjacency.

Node Embeddings

Each node i carries a 384-dimensional feature vector extracted by the DINO/ViT teacher encoder (Section S2). The spatial coordinates of each tile within the WSI define node positions.

Adjacency Matrix

Edges were defined using a k-nearest-neighbour (KNN) criterion with an additional distance threshold. The adjacency matrix A is defined as:

where KNN(i) denotes the set of k nearest neighbours of node i (based on Euclidean distance in tile coordinate space), D(i, j) is the Euclidean distance between nodes i and j, and d is a distance threshold. This construction ensures that each node is connected to its local spatial neighbourhood while preventing spurious long-range edges across tissue gaps or artefacts. The resulting graph is sparse and reflects the contiguous spatial architecture of the tumour and its microenvironment.

Section S4. GIN + CNN Neural Network Architecture

Both the CSDI model and the Good Prognosis Class Prediction model share a common GIN+CNN architecture. This design enables effective extraction of graph-level representations from the spatial tile graphs described in S3.

Graph Isomorphic Network (GIN) Component

The GIN layers aggregate node features from local neighbourhoods using a sum aggregation scheme. After message passing through the GIN layers, graph-level representations are obtained by sum pooling and concatenating node embeddings across all nodes in the graph. This produces a fixed-length graph embedding that captures both local tile-level features and global graph topology.

Convolutional Neural Network (CNN) Component

Because a WSI graph may contain hundreds to thousands of nodes, a follow-on CNN is applied to the graph embedding to extract higher-order features and reduce dimensionality. This design allows the model to handle graphs of variable size while producing a fixed-dimensional patient-level representation.

Clinical Variable Integration

A selection of clinical variables — age, sex, ethnicity, pT stage, pN stage, and tumour site — are encoded as high-level embeddings and concatenated into the fully-connected layer of the CNN component, enabling the model to integrate clinical context with WSI-derived morphological information.

Section S5. Cancer-Specific Death Index (CSDI) Model

The CSDI model performs lifetime analysis on the spatial WSI graph to predict disease-specific survival (DSS). Cox proportional hazards (CPH) modelling is used to link graph-level features to DSS time.

Cox Proportional Hazards Framework

We use cox proportional hazards models (CPH)[28] to evaluate disease specific survival (DSS) time. A general hazard function (t|x) represents the risk of dying at time t can be estimated as follow:

$$\lambda(t|x) = \lambda_0(t)e^{h(x)} \tag{S1}$$

where $\lambda_0(t)$ is the baseline hazard function and h(x) is the log-relative hazard estimated by the GIN+CNN network, following the general deep Cox regression paradigm [30]. The CPH model is semi-parametric: it estimates h(x) without requiring specification of $\lambda_0(t)$.

CSDI Formulation

A Cancer-Specific Death Index (CSDI) is defined by applying a sigmoid transformation to h(x), bounding the output to [0, 1]:

$$CSDI(x) = \frac{1}{1+e^{-\alpha\cdot h(x)+\beta}} \quad \text{(S2)}$$

where α and β are real-valued learnable parameters. Defining. $c(x) = \log\left(\frac{1}{CSDI(x)} - 1\right)$, and setting α′ = −1/α and β′ = β/α, Equation S2 simplifies to:

$$h(x) = \alpha' c(x) + \beta' \quad \text{(S3)}$$

This formulation preserves the monotonic relationship between CSDI and the hazard ratio while avoiding numerical instability in deep network training.

Loss Function

The model is optimised using the average negative log partial likelihood loss. Applying Equation S3:

$$L = -\frac{1}{N_{E=1}}\sum_{i:E_i=1}(\alpha'\widehat{c(x_i)} + \beta' - log(\sum_{j\in R(T_i)} e^{\alpha'\widehat{c(x_j)}+\beta'})) \quad \text{(S4)}$$

where:

- $N_{E=1}$ is the number of patients who experienced a cancer-specific death event
- E_i = 1 if patient i experienced a cancer-specific death event, 0 otherwise
- T_i is the time of the cancer-specific death event for patient i
- $R(T_i)$ is the risk set: patients who had not experienced a cancer-specific death event before time T_i
- x_i is the WSI spatial graph feature vector for patient i
- $\hat{c}(x_i)$ is the model's predicted $c(x_i)$

Section S6. Good Prognosis Class Prediction Model

The Good Prognosis Class Prediction model uses the same GIN+CNN architecture as the CSDI model (Section S4) but is formulated as a binary classification task: the model outputs a probability that a patient belongs to the good prognosis (low-risk) class.

Class Imbalance and Focal Loss

In the training dataset, good prognosis patients substantially outnumber poor prognosis patients, creating class imbalance that can bias a standard cross-entropy classifier. Focal loss (FL) [29] was used to mitigate this by down-weighting the loss contribution of well-classified easy examples and focusing training on hard, misclassified cases:

$$FL(p_t) = -(1-p_t)^{\gamma}\log(p_t) \quad \text{(S5)}$$

where p_t is the model's estimated probability for the true class, and $\gamma > 0$ is a focusing parameter. Setting $\gamma > 0$ reduces the relative loss for well-classified examples ($p_t > 0.5$) and concentrates gradient updates on challenging misclassified cases. The value of γ was tuned on the QUASAR2 validation set.

Section S7. Final Risk Classification and Threshold Determination

The CSDI model and the Good Prognosis Class Prediction model were combined in an ensemble framework. The final three-tier risk assignment for each patient was determined as follows:

- Low risk (good prognosis): CSDI ≤ T_{low} AND Good Prognosis Class Prediction = positive
- High risk (poor prognosis): CSDI ≥ T_{high}
- Intermediate risk: all remaining patients

The two threshold values T_{low} and T_{high} were selected using the pooled out-of-fold predictions from the five-fold cross-validation on the development dataset: for each threshold, candidate values were evaluated on the negative predictive value (NPV) of the corresponding risk classification (low-risk for T_{low}, high-risk for T_{high}), and the value maximising sensitivity subject to an NPV of at least 90% was selected, further constrained so that approximately 80% of patients were allocated to the low-risk group and approximately 10% to the high-risk group. This procedure reflects the clinical priority of confidently ruling out disease-specific death in the low-risk group while keeping the high-risk group small and enriched for true positives. Thresholds were fixed after this optimisation step and were not modified during external validation on TCGA-COAD or SCOT.

The ensemble design is intentional: requiring concordance between a survival-regression signal (CSDI) and a binary classification signal (Good Prognosis Class) for low-risk designation increases specificity for the good prognosis call, which is the highest-stakes clinical decision (identifying patients who can safely forgo adjuvant chemotherapy).

Section S8. GCN Surrogate Model for Spatial Interpretability

To generate spatially interpretable risk maps, a Graph Convolutional Network (GCN) combined with Cox proportional hazards modelling was trained as a surrogate model in parallel to the primary GIN+CNN model. Both models operate on the same spatial tile graph; however, the GCN surrogate is specifically designed to produce a tile-level Cancer-Specific

Death Index (CSDI) for each node (tile), quantifying its local contribution to overall prognostic risk. This approach provides stable, gradient-free spatial attribution and was adopted following discontinuation of Grad-CAM, which exhibited instability in the graph-based architecture.

Computation

For each node n in the spatial tile graph, the GCN surrogate model produces a node-level CSDI score $S^{GcN}(n)$ by propagating neighbourhood-aggregated features through graph convolutional layers and applying a Cox proportional hazards output head. This forward-pass attribution is computationally efficient and avoids the gradient instability associated with backpropagation-based methods. The per-node scores directly reflect the local morphological contribution to the patient-level prognostic risk estimate.

Normalisation and Visualisation

Node scores are normalised to [0, 1] across all tiles in the WSI. A false-colour scheme (e.g., blue–green–yellow–red, low to high contribution) is applied to the normalised scores to generate a heatmap image. This heatmap is overlaid on the original H&E-stained WSI tile grid, enabling pathologists to identify which morphological regions — whether tumour nests, stromal compartments, or immune infiltrates — drove the CSDI risk prediction for each individual patient.

Section S9. Cross-Validation and External Validation Protocol

Internal Validation — Five-Fold Cross-Validation on QUASAR2

Prior to model development, approximately 20% of the QUASAR2 cohort (N = 225) was reserved as a locked holdout test set. For the purposes of this stratified split, each patient's risk group was a ground-truth label derived directly from their observed disease-specific survival (DSS) outcome and time-to-event data — computed independently of, and prior to, any model training or prediction — not a model-predicted risk classification. A stratified sampling algorithm was applied to ensure that each ground-truth risk group was proportionally represented: 20.64%, 19.78%, and 20.15% of the low-, high-, and intermediate-risk patients, respectively, were assigned to the holdout set. The remaining 80% of the QUASAR2 cohort with evaluable DSS outcomes constituted the development dataset.

Five-fold cross-validation (CV) was performed on the development dataset for model training, hyperparameter selection, and threshold determination. In each fold, four-fifths of the development data were used for training and one-fifth for evaluation, maintaining risk-

group stratification throughout. Table S1 shows the percentage of each risk group allocated to each subset across all five folds and the holdout test set.

Table S1. Risk-group-stratified data allocation across five-fold cross-validation folds and holdout test set (% of each risk group).

Fold	Subset	Low Risk (%)	High Risk (%)	Intermediate Risk (%)
CV0	Train	63.54	62.64	64.62
	Evaluation	15.82	17.58	15.23
CV1	Train	63.27	65.93	63.85
	Evaluation	16.09	14.29	16.00
CV2	Train	63.27	63.74	63.54
	Evaluation	16.09	16.48	16.31
CV3	Train	63.54	64.84	63.69
	Evaluation	15.82	15.38	16.15
CV4	Train	63.81	63.74	63.70
	Evaluation	15.55	16.48	16.15
Holdout Test	—	20.64	19.78	20.15

Performance metrics were averaged across all five folds to obtain robust estimates and assess variance. Thresholds T_{low} and T_{high} (Section S7) were fixed on the aggregated evaluation fold results before assessing holdout test set performance.

External Validation — TCGA-COAD and SCOT

The TCGA-COAD (n=248) and SCOT (n=671) datasets served as independent external validation cohorts. Neither cohort was used at any stage of model training, hyperparameter selection, or threshold determination. The final trained model (weights fixed after QUASAR2 five-fold CV) was applied directly to both cohorts in inference mode. SCOT in particular, as a fully independent international multicentre trial cohort, provides the strongest evidence of generalisability across patient populations, pathology laboratory practices, and digital scanning environments.

Note: TCGA-COAD was used for additional model development (confirmation of learned representations) but not for threshold calibration. The primary claims regarding clinical utility rest on the SCOT hold-out validation.

Section S10. Model Training Results

Implementation Details

All model training was performed on shared IBM AC922 compute infrastructure (32× IBM POWER9 CPU cores, 512GB DDR4 RAM, 4× NVIDIA Tesla V100 GPUs; RHEL

8), using Python 3.10, PyTorch 2.0.1, CUDA 12.1, and — for the graph neural network component of Stage 2 — the Deep Graph Library (DGL) 2.2.1.

Stage 1 (DINO/ViT self-supervised encoder): trained using the AdamW optimiser (β = (0.9, 0.999); weight decay 5×10^{-4}; AMSGrad disabled) with a linear learning-rate warm-up over the first 10 epochs, ramping to a peak learning rate of 5×10^{-4} at the end of warm-up, followed by cosine annealing down to a target learning rate of 1×10^{-6} by the end of training. Training was run for 100 epochs, with a batch size of 64 per GPU; Stage 1 training typically used 3 of the 4 available GPUs, with the remaining GPU reserved for concurrent lightweight training/inference tasks (occasionally all 4 GPUs were used when available). Stage 2 (GIN+CNN survival model): trained using the Adam optimiser (base learning rate 1×10^{-3}; β = (0.9, 0.999); weight decay 0; AMSGrad disabled). Training was run for 100 epochs, with a training and validation batch size of 192.

Hyperparameter optimisation for the DINO self-supervised Vision Transformer (ViT) feature extraction model was conducted through systematic experimentation, with results summarised in Figure S1. As shown in Figure S1A, models trained without a positional encoder exhibited markedly higher training C-index values (range 0.830–0.852) relative to their corresponding validation C-index values (range 0.596–0.612), a pattern consistent with overfitting attributable to loss of spatial context in tile feature representations. Incorporation of a positional encoder substantially reduced this train–validation gap, with validation C-index improving to the range 0.622–0.635 despite lower training C-index values (0.709–0.783). A grid search over gradient clipping threshold (clip-grad: 1–5) and attention drop rate (0, 0.1, 0.15) showed that clip-grad = 3.0 with attention drop rate = 0.1 yielded the highest validation C-index of 0.645 (Figure S1B); differences across the tested range were modest, suggesting the model is relatively robust to moderate regularisation choices. A systematic grid search over transformer depth (4, 8, 12, and 16 layers) and number of attention heads (3, 4, 6, and 8) is shown in Figures S1C1 and S1C2. The combination of depth L = 12 and six attention heads yielded the highest values for both training C-index (0.773; Figure S1C1) and validation C-index (0.663; Figure S1C2), confirming this configuration as optimal. Notably, this configuration also exhibited the smallest train–validation C-index gap among all depth-12 configurations (0.110), indicating relatively strong generalisation at the selected architecture. Based on these

experiments, the following hyperparameters were selected for the final ViT encoder: depth L = 12, number of attention heads = 6, feature dimensionality = 384, gradient clipping threshold (clip-grad) = 3.0, and attention drop rate = 0.1.

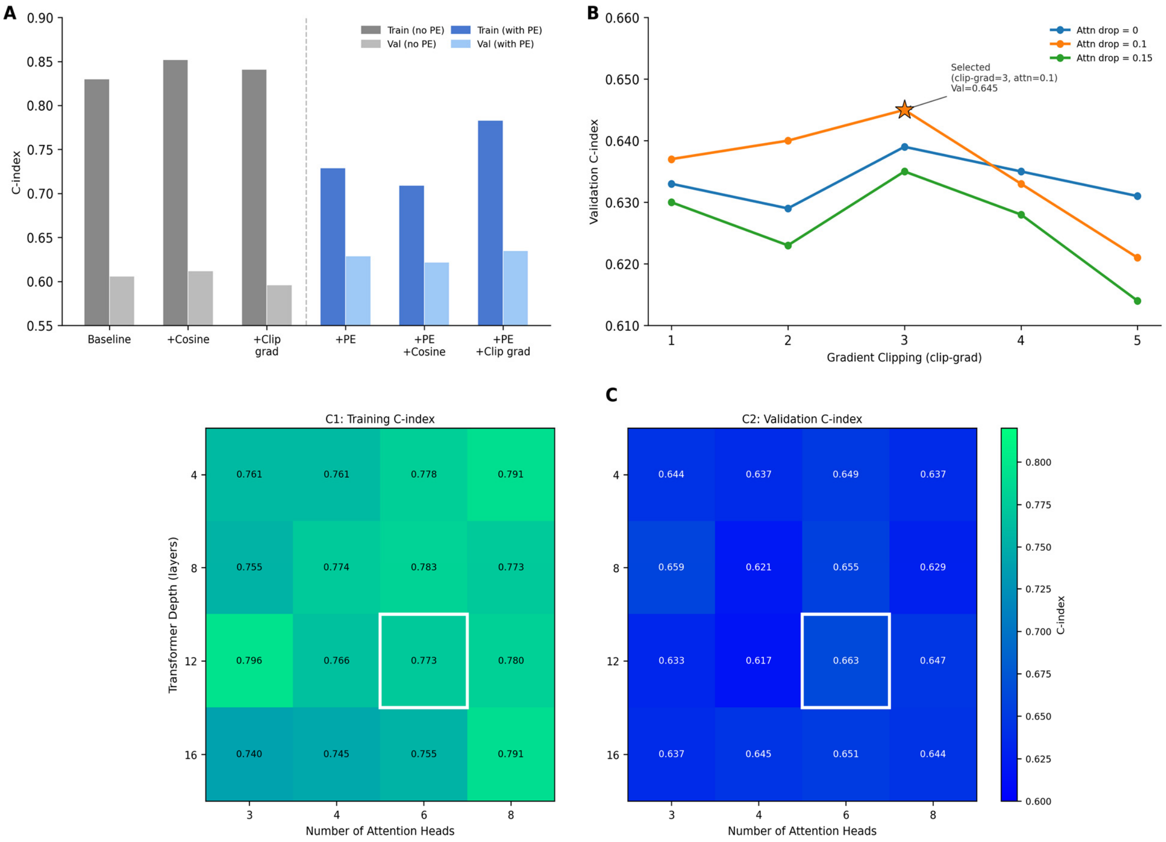

Figure S1. ViT Hyperparameter Optimisation for the DINO Self-Supervised Feature Extractor. (A) Ablation study showing paired training (dark bars) and validation (light bars) C-index for each configuration; the large train–validation gap in models without a positional encoder (grey) indicates overfitting, which is substantially reduced upon its inclusion (blue). (B) Validation C-index across a grid search of gradient clipping threshold (clip-grad: 1–5) and attention drop rate (0, 0.1, 0.15); the selected configuration (clip-grad = 3.0, attention drop = 0.1; star) achieved the highest validation C-index of 0.645. (C1) Training C-index and (C2) Validation C-index across a grid search of transformer depth (4–16 layers) and number of attention heads (3–8); both panels share the same colour scale to allow direct visual comparison of the train–validation gap. The selected configuration (depth L = 12, heads = 6; white border) achieves the highest validation C-index of 0.663 and the smallest train–validation gap among depth-12 configurations.

Stage 2 GIN+CNN model training was conducted on the QUASAR2 development dataset (N=892, 80% of the 1,117 patients with evaluable DSS outcomes) using five-fold cross-validation, with the ViT encoder weights frozen at the configuration identified in Stage 1. Tile resolution and Monte Carlo random tile subsampling ratio were evaluated concurrently across three configurations. At 10× magnification (no subsampling), the mean five-fold Training C-index was 0.587 (SD 0.030; range 0.548–0.624) and the mean Validation C-index was 0.523 (SD 0.015; range 0.507–0.545). Performance improved at 20× magnification with a 1/16 Monte Carlo

sampling ratio (mean Training C-index 0.625, SD 0.028, range 0.583–0.654; mean Validation C-index 0.568, SD 0.014, range 0.544–0.579), and was highest at 40× with a 1/32 Monte Carlo sampling ratio (mean Training C-index 0.649, SD 0.037, range 0.596–0.687; mean Validation C-index 0.592, SD 0.039, range 0.559–0.659). The 40× configuration with 1/32 Monte Carlo tile subsampling was therefore selected as the final model.

To evaluate whether HistoNav's prognostic performance was consistent across AJCC stage subgroups, Kaplan–Meier survival curves stratified by HistoNav risk group were generated separately for Stage II and Stage IIIA patients, pooled across all three cohorts (QUASAR2, TCGA-COAD, SCOT) (Figure S2). For Stage II patients (n=479), five-year DSS was 93.4% (95% CI: 90.2%–95.6%) for low-risk (n=435), 87.8% (95% CI: 66.7%–95.9%) for intermediate-risk (n=30), and 64.2% (95% CI: 30.2%–84.8%) for high-risk (n=14) patients, with a hazard ratio for high- versus low-risk of HR = 6.061 (95% CI: 2.124–17.298; log-rank P = 0.006691). For Stage IIIA patients (n=392), five-year DSS was 93.0% (95% CI: 88.5%–95.7%) for low-risk (n=265), 82.8% (95% CI: 68.0%–91.2%) for intermediate-risk (n=62), and 70.9% (95% CI: 55.9%–81.6%) for high-risk (n=65) patients, with HR = 4.093 (95% CI: 2.200–7.612; log-rank P = 0.000045). Prognostic separation between risk groups was therefore maintained within both stage subgroups, with the wider confidence intervals in the Stage II high-risk group (n=14) reflecting its smaller sample size.

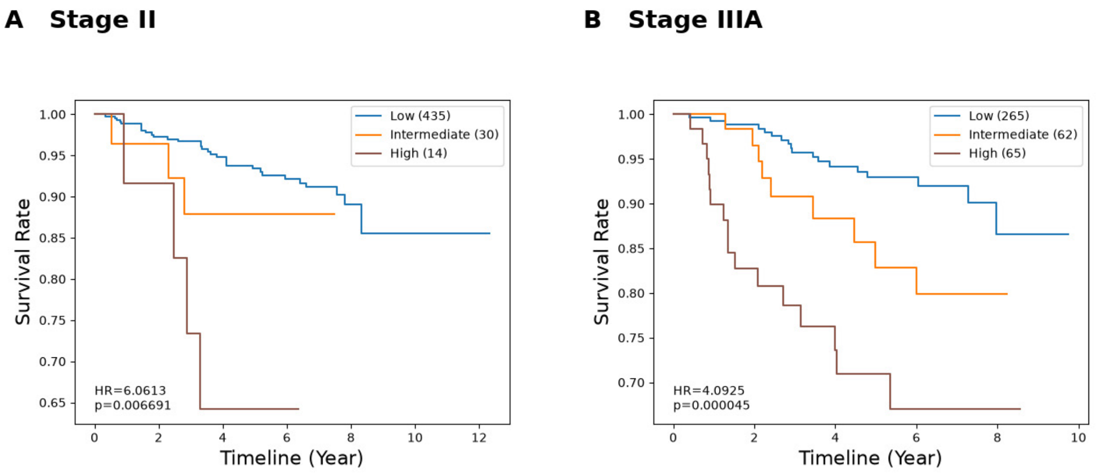

Figure S2. *Kaplan–Meier Disease-Specific Survival Curves by HistoNav Risk Group, Stratified by AJCC Stage. Kaplan–Meier curves for disease-specific survival (DSS) stratified by HistoNav risk classification, shown separately for (A) Stage II (n=479: low-risk n=435, intermediate-risk n=30, high-risk n=14; HR high vs. low = 6.061, 95% CI: 2.124–17.298, log-rank P = 0.006691) and (B) Stage IIIA (n=392: low-risk n=265, intermediate-risk n=62, high-risk n=65; HR = 4.093, 95% CI: 2.200–7.612, P = 0.000045) patients, pooled across all three cohorts (QUASAR2, TCGA-COAD, SCOT).*

To provide a more comprehensive evaluation of model performance over time, time-dependent (incident/dynamic) AUC was estimated at 1, 3, and 5 years using inverse probability of censoring weighting (IPCW), implemented via the cumulative_dynamic_auc() function from the scikit-survival (sksurv) Python package, applied to the pooled cohort. Time-dependent AUC was 0.726 (95% CI: 0.556–0.878) at 1 year, 0.720 (95% CI: 0.603–0.866) at 3 years, and 0.711 (95% CI: 0.632–0.788) at 5 years (Figure S3), indicating stable discriminative performance over the follow-up period, consistent with the AUC of 0.72 reported for low-risk classification in the main manuscript.

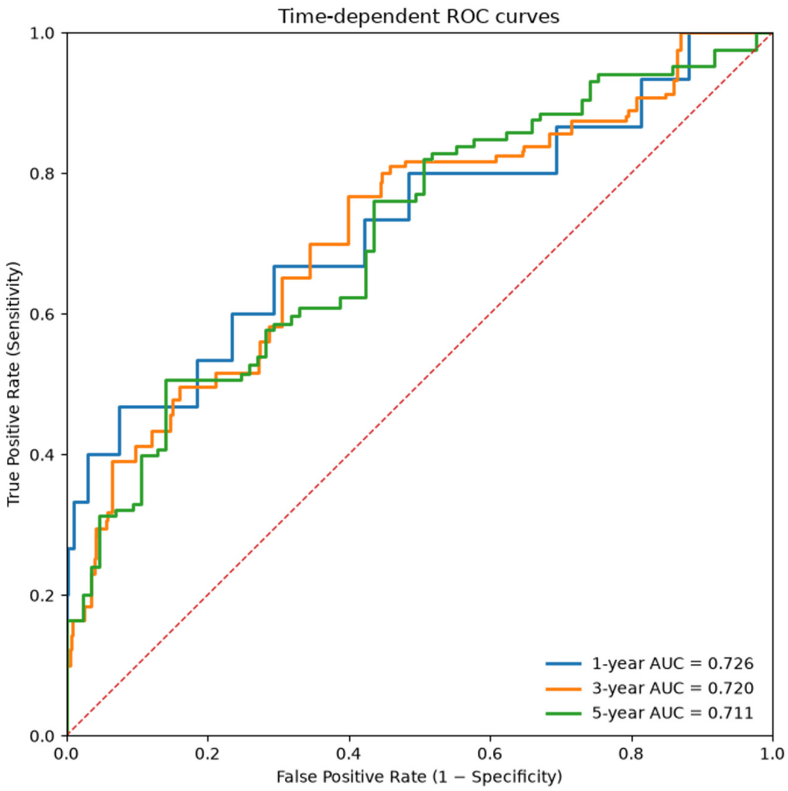

Figure S3. *Time-Dependent (Incident) Receiver Operating Characteristic Curves at 1, 3, and 5 Years. Time-dependent ROC curves and AUC estimated using inverse probability of censoring weighting (IPCW) via scikit-survival's cumulative_dynamic_auc() function, applied to the pooled cohort (N=871): 1-year AUC = 0.726 (95% CI: 0.556–0.878), 3-year AUC = 0.720 (95% CI: 0.603–0.866), 5-year AUC = 0.711 (95% CI: 0.632–0.788). The red dashed line indicates chance-level discrimination (AUC = 0.5).*

Section S11. Extended Discussion: Additional Robustness and Comparator Considerations

S11.1 Robustness to H&E Imaging Variability

Variability in H&E imaging conditions — encompassing differences in staining protocols, scanner hardware, tissue preparation, and inter-laboratory workflow — is a well-recognised challenge in deploying deep learning models for computational pathology. As documented in a recent comprehensive survey of deep learning methods for histopathological image analysis, variations between pathology laboratories, between technicians, and across different batches of reagents can induce substantial differences in colour saturation, contrast, and image resolution that materially degrade model performance [46]. HistoNav addresses this challenge at the architectural level: the DINO self-supervised Vision Transformer feature extractor is trained without tile-level annotations and learns morphological representations that are inherently less sensitive to staining intensity and scanner-specific colour rendition than supervised alternatives. The empirical per-cohort performance data presented in Table 2 of the main manuscript provide direct evidence of this robustness: the clinically critical NPV (~0.96) and specificity for low-risk classification (~0.88–0.94) remained consistent across three cohorts acquired at different institutions, on different scanner platforms, and spanning over a decade of sample collection, confirming that HistoNav's prognostic signal is not confounded by inter-site imaging variation.

S11.2 Extended Comparison with Molecular Prognostic Signatures

Several validated molecular prognostic signatures have been proposed for Stage II CRC, but each carries significant practical constraints that limit scalability in routine clinical pathology. ColoPrint, an 18-gene microarray classifier, identifies approximately 60–73% of Stage II patients as low-risk with 5-year recurrence rates of 10–12%, and has been shown to outperform ASCO clinical risk criteria in identifying patients who may safely avoid adjuvant chemotherapy [38,39]. The Oncotype DX Colon Recurrence Score, a 12-gene RT-PCR assay validated across real-world populations exceeding 20,000 patients, generates continuous risk scores that correlate with recurrence probability but requires proprietary commercial processing [40]. CDX2, a homeobox transcription factor detectable by immunohistochemistry, is lost in approximately 4% of Stage II colon tumours; its absence confers substantially worse prognosis (5-year DFS 49–51% versus 80–87% in CDX2-positive patients, HR 3.44) and identifies a subgroup who appear to derive particular benefit from adjuvant fluoropyrimidine-based chemotherapy [41,42]. While these signatures represent meaningful advances in prognostic precision, they require specialised molecular assays, proprietary reagents, or ancillary immunostaining beyond standard diagnostic workup, and operate on single molecular dimensions without capturing the spatial and architectural tissue heterogeneity increasingly recognised as prognostically relevant. HistoNav, by contrast, extracts rich spatial morphological features from routine H&E slides already produced as part of standard-of-care pathology, applies to the complete patient

population without additional testing, and integrates architectural context through graph-based modelling of tissue spatial relationships. Consistent with this, MSI/MMR status alone was not significantly prognostic for disease-specific survival in the QUASAR2 cohort (HR=1.19, 95% CI 0.74–1.92, p=0.465; n=772), underscoring the limitations of single molecular biomarkers for risk stratification in this population.

References for Supplementary Methods

Numbers correspond to the main manuscript reference list. Key references for technical components:

- [24] Chen LC et al. Encoder-Decoder with Atrous Separable Convolution for Semantic Image Segmentation. ECCV 2018.
- [25] Macenko M et al. A method for normalizing histology slides for quantitative analysis. ISBI 2009.
- [26] Caron M et al. Emerging Properties in Self-Supervised Vision Transformers. ICCV 2021.
- [27] Dosovitskiy A et al. An Image is Worth 16×16 Words: Transformers for Image Recognition at Scale. ICLR 2021.
- [28] Cox DR. Regression Models and Life-Tables. J R Stat Soc B. 1972;34:187–220.
- [29] Lin TS et al. Focal Loss for Dense Object Detection. IEEE TPAMI. 2020;42(2):318–327.
- [30] Katzman JL et al. DeepSurv: personalised treatment recommender system using a Cox proportional hazards deep neural network. BMC Med Res Methodol. 2018;18:24.
- [46] Qin L et al. A Survey of Deep Learning in Histopathological Nuclear Segmentation. IEEE Comput Intell Mag. 2025;20:19–40.
